# The Timing of Nitrogen Addition Impacts Yeast Genes Expression and the Production of Aroma Compounds During Wine Fermentation

**DOI:** 10.3389/fmicb.2022.829786

**Published:** 2022-02-22

**Authors:** Joséphine Godillot, Isabelle Sanchez, Marc Perez, Christian Picou, Virginie Galeote, Jean-Marie Sablayrolles, Vincent Farines, Jean-Roch Mouret

**Affiliations:** ^1^SPO, INRAE, L’Institut Agro Montpellier, Université de Montpellier, Montpellier, France; ^2^MISTEA, INRAE, L’Institut Agro Montpellier, Montpellier, France

**Keywords:** wine, alcoholic fermentation, nitrogen, fermentative aromas, Box-Behnken design, transcriptomic analysis

## Abstract

Among the different compounds present in the must, nitrogen is an essential nutrient for the management of fermentation kinetics but also plays an important role in the synthesis of fermentative aromas. To address the problems related to nitrogen deficiencies, nitrogen additions during alcoholic fermentation have been implemented. The consequences of such additions on the main reaction are well known. However, their impact on aromas synthesis is still poorly understood. So, the main objective of this study was to determine the impact of nitrogen addition during the stationary phase on both the fermentation kinetics and aroma synthesis. To reach this goal, we used a transdisciplinary approach combining statistical modeling (Box-Behnken design and response surface modeling) and gene expression study (transcriptomic analysis). Our results indicated that nitrogen metabolism, central carbon metabolism (CCM), fermentation kinetics and aroma production were significantly impacted by nitrogen addition. The most remarkable point was the different regulation of the bioconversion of higher alcohols into acetate esters on one hand and of fatty acids into ethyl esters on the other hand. We highlighted that the conversion of higher alcohols into acetate esters was maximum when nitrogen was added at the beginning of the stationary phase. Conversely, the highest conversion of acids into ethyl esters was reached when nitrogen was added close to the end of the stationary phase. Moreover, even if the key element in the production of these two ester families appeared to be the enzymatic activity responsible for their production, rather than the availability of the corresponding precursors, these enzymatic activities were differently regulated. For acetate esters, the regulation occurred at gene level: the *ATF2* gene was overexpressed following nitrogen addition during the stationary phase. On the opposite, no induction of gene expression was noted for ethyl esters; it seemed that there was an allosteric regulation.

## Introduction

In recent years, consumer interest in fruity wines has been growing steadily. The main actors of the fruity organoleptic properties are the aromas produced during the fermentation process. Among the different volatile compounds produced by the yeast, esters (acetate and ethyl esters) are the compounds mostly responsible for fruity aromas of young wines (Swiegers et al., 2005; Ugliano et al., 2010). The synthesis of these different aromas depends in part on the yeast strain (Massoutier et al., 1998; Lambrechts and Pretorius, 2000), the composition of the must (Rollero et al., 2015), the fermentation temperature (Molina et al., 2007; Morakul et al., 2011; Mouret et al., 2014a), but is also highly impacted by nitrogen, which is the limiting nutrient for yeast growth in enological conditions (Bely et al., 1990b; Mouret et al., 2014a; Gobert et al., 2019).

Nitrogen in organic (amino acids) or mineral (ammonium) forms is already naturally present in the grape must, but extra nitrogen is sometimes added to overcome the problem of slow fermentations (Salmon, 1989; Bely et al., 1990b; Varela et al., 2004). Moreover, many studies have shown that an addition of nitrogen at the start of the fermentation has important effects on the synthesis of fermentative aromas (Rollero et al., 2015; Gobert et al., 2019). Production of higher alcohols as a function of nitrogen content is not linear: below a threshold value (from 200 to 300 mgN/L), alcohol synthesis increases with nitrogen content, but above this value, alcohol production decreases (Vilanova et al., 2007, 2012; Rollero et al., 2015). The behavior of propanol is very different from that of the other higher alcohols; it can be considered as a marker of assimilable nitrogen availability (Mouret et al., 2014b) as its synthesis is related to the quantity and quality of available nitrogen. Its maximum concentration is reached when all nitrogen has been consumed by the yeast at the end of the growth phase corresponding to around 30% sugar consumption (Mouret et al., 2014b; Seguinot et al., 2018) whereas the other higher alcohols are continuously synthesized throughout the fermentation process (Mouret et al., 2014b). Conversely, ester production is directly correlated to and increases with the initial nitrogen content (Hernandez-Orte et al., 2006; Torrea et al., 2011; Rollero et al., 2015).

Research works have also been carried out to evaluate the impact of nitrogen additions performed during the stationary phase. These additions have been shown to be extremely effective for fermentation kinetics on one hand, since nitrogen will reduce the fermentation time (Bely et al., 1990a; Malherbe et al., 2004), but on the other hand, these additions also impact aroma production (Hernandez-Orte et al., 2006; Jiménez-Martí et al., 2007; Seguinot et al., 2018). Indeed, the effect of such an addition on acetate esters is very significant and consistently increases their concentration. Seguinot et al. (2018) showed that the concentration of isoamyl acetate increased from 1.5 mg/L to 4 mg/L after ammonium addition during the stationary phase. Propanol synthesis is also highly stimulated following an inorganic nitrogen addition whereas the concentration of the other higher alcohols remains unchanged (Seguinot et al., 2018).

While the effect of the initial nitrogen addition has been well explored, little has been done regarding the impacts of the timing and of the quantity of nitrogen added during the stationary phase. So, to get a deeper understanding of the effect of these nitrogen additions, we implemented a multidisciplinary approach combining bioprocess management, aroma analysis and study of gene expression. A Box-Behnken experimental design was used to evaluate the direct and interaction effects of three parameters: initial nitrogen concentration, added nitrogen concentration and timing of nitrogen addition during the stationary phase. The effect of these three parameters on fermentation kinetics and aroma production was analyzed. A complementary transcriptomic analysis was also performed to understand in more details nitrogen effect on yeast metabolism.

## Materials and Methods

### Yeast Strain

The *Saccharomyces cerevisiae* strain used in this study is the commercial strain Lalvin EC-1118 (Lallemand SA, Montreal, Canada). Fermentation tanks were inoculated with 10 g/hL active dry yeast previously rehydrated for 30 min at 37°C in a 50 g/L glucose solution.

### Fermentation Media

All fermentations were carried out in synthetic musts simulating a standard grape juice (Bely et al., 1990a). The detailed compositions of the different stock solutions used for the preparation of this synthetic medium are identical to the ones presented in Mouret et al. (2014b). All musts contained 180 g/L of sugars (90 g/L glucose and 90 g/L fructose) and their pH was 3.3. Three initial assimilable nitrogen (composed of ammonium chloride and mixture of amino acids) concentrations were tested: 70, 140, and 210 mgN/L, respectively, corresponding to media SM70, SM140, and SM210. The proportions of the different sources were identical in all three media.

### Fermentation Conditions

The fermentations were performed in 1.2 L glass fermenters with 1 L of medium at 24°C. CO_2_ release was accurately measured from an automatic online monitoring of weight loss (Sablayrolles et al., 1987).

Different concentrations – 50, 100, and 150 mgN/L – of mineral nitrogen (di-ammonium phosphate) were added at different timings of the stationary phase: 20, 35, and 50 g/L of produced CO_2_ (corresponding, respectively, to 24, 42, and 60% of fermentation progress). Nitrogen additions were performed automatically by means of a pump on which a syringe containing the nitrogen solution was placed.

The data summarizing the fermentation kinetics are stored in the ALFIS database^1^. Thanks to this database and the use of a custom R program, we retrieved the key points of the fermentation kinetics (Vmax1, dVmax, and Fermentation time).

### Cell Population

During fermentation, the total cell population was determined using a Beckman Coulter counter (Model Z2, Beckman-Coulter, Margency, France) fitted with a 100 μm aperture probe.

### Measurement of Assimilable Nitrogen

Ammonium concentration was determined enzymatically (R-Biopharm, Darmstadt, Germany). The free amino acid content was determined by cation exchange chromatography, with post-column ninhydrin derivatization (Biochrom 30, Biochrom, Cambridge, United Kingdom) as described by Crépin et al. (2012).

### Determination of Metabolite Compounds

Ethanol, glycerol, succinate, and acetate concentrations were determined by HPLC (HPLC 1290 Infinity, Agilent Technologies, Santa Clara, CA, United States) in a Phenomenex Rezex ROA column (Agilent Technologies, Santa Clara, CA, United States) at 60 C. The column was eluted with 0.005 N H_2_SO_4_ at a flow rate of 0.6 mL/min. The acetate concentration was determined with a UV meter at 210 nm; the concentrations of the other compounds were determined with a refractive index detector. Analysis was carried out with the Agilent EZChrom software package.

### Analysis of Volatile Compounds

The analysis of fermentative aromas was performed following the method described by Rollero et al. (2015). First, the volatile compounds were extracted with dichloromethane. Then, the concentrations of fermentative aromas were measured via GC/MS in SIM mode using a DB-WAX GC column. Thirty-five compounds were quantified using internal deuterated standards.

### Experimental Design

Statistical analysis was performed with R software, version 4.0.2 (R Development Core Team, 2012) and the rsm library (Lenth, 2009).

A Box-Behnken experimental design was applied to investigate the effects of three independent variables (initial nitrogen, timing and concentration of nitrogen addition) and calculate the optimal combination of treatment conditions for fermentation kinetics, the production of central carbon metabolism (CCM) metabolites and volatile compounds, with a limited number of experimental runs. A total of 16 experiments were performed, including four at the center of the experimental domain. In this experimental design, there were three coded factor levels: −1, 0, +1 in which −1 corresponded to the low level of each factor, +1 to the high level, and 0 to the mid-level. The actual level of each factor was calculated with the following equation (Eq. 1) (Box and Behnken, 1960):


$$\mathrm{Actual}\,\mathrm{level}=\mathrm{Coded}\,\mathrm{value}\,x\,\frac{\mathrm{High}\,\mathrm{value}-\mathrm{low}\,\mathrm{value}}{2}+\frac{\mathrm{High}\,\mathrm{level}-\mathrm{low}\,\mathrm{level}}{2}$$

The effect of the three independent variables on each measured parameter (Y) was modeled with a polynomial response surface (Eq. 2):


$$Y=\beta_{0}+\beta_{1}\,x_{1}+\beta_{2}\,x_{2}+\beta_{3}\,x_{3}+\beta_{12}\,x_{1}\,x_{2}+\beta_{13}\,x_{1}\,x_{3}+\beta_{23}\,x_{2}\,x_{3}+\beta_{11}\,x_{1}^{2}+\beta_{22}\,x_{2}^{2}+\beta_{33}\,x_{3}^{2}+\varepsilon$$

Where x1, x2, and x3 represent the coded values of initial nitrogen content, timing of nitrogen added and concentration of nitrogen added, respectively, Y is the predicted response, β_0_ the intercept term, β_i_ the linear coefficient, β_ii_ the quadratic coefficient, and β_ij_ the interaction coefficient (*i* = 1, 2, and 3) and ε are independent N (0, σ^2^) distributed error terms. When necessary, a simplified model was fitted for some compounds by suppressing the interactive terms of the equation according to validity criteria. In addition, the normality of residual distributions and homogeneity of variance were studied with standard diagnostic graphs; no violation of the assumptions was detected. The accuracy and general ability of each polynomial model described above was evaluated by a lack of fit test, the Fisher test and the adjusted coefficient of determination adjR^2^. The reliability of the fitted models was overall very good: each polynomial model gave a non-significant lack of fit test at a 0.05 threshold, a significant Fisher test at a 0.05 threshold and a range of adjR^2^ between 57 and 99%. For graphical representations of the surface responses, a custom version of the persp() function was used.

### RNA Extraction and Microarray Assay

Two types of fermentations were performed to study the expression of yeast genes following nitrogen addition (150 mgN/L of added nitrogen). The first was conducted with an addition at 20 g/L of produced CO_2_ and the second with an addition at 50 g/L of produced CO_2_. These fermentations were carried out with 140 mgN/L initial assimilable nitrogen and were performed in triplicate. Cells were harvested 2 h after nitrogen additions. We also implemented a control without nitrogen addition to better understand its impact on gene expression. To be able to compare the conditions with/without additions in the most accurate way, we took samples from the control according to the fermentation progress and not according to the time after additions.

The total RNA extraction and microarray assay protocols were performed with the method used by Rollero et al. (2016) and Duc et al. (2017).

### Statistical Analysis of Microarray Data

The R4.0.2 software was used for statistical analyses.

The limma package was used to import and normalize the overall microarray data (quantile method for interarray normalization). The dataset is available in the Gene Expression Omnibus Database (GSE189010). To determine the differential gene expression between experimental conditions, a modified *t*-test was performed by filtering on confidence at *p* < 0.05, using the Benjamini and Hochberg false discovery rate as multiple testing corrections of the *t*-test *p*-values. The genes with different levels of expression were grouped according to gene ontology (GO) process terms using the GeneCodis program (García-Moreno et al., 2021)^2^.

## Results

We investigated the cross-impacts of the initial nitrogen concentration (N0), the timing of nitrogen addition (Tm) and the concentration of added nitrogen (Nad) during the stationary phase on fermentation kinetics, central carbon metabolism, aromas production and yeast gene expression.

Nitrogen and sugars were exhausted at the end of all fermentations. Final concentrations of ethanol were identical in all the conditions.

### Effects of Nitrogen Addition on Fermentation Kinetics, Cell Count, and Metabolites of the Central Carbon Metabolism

**FIGURE 1:**
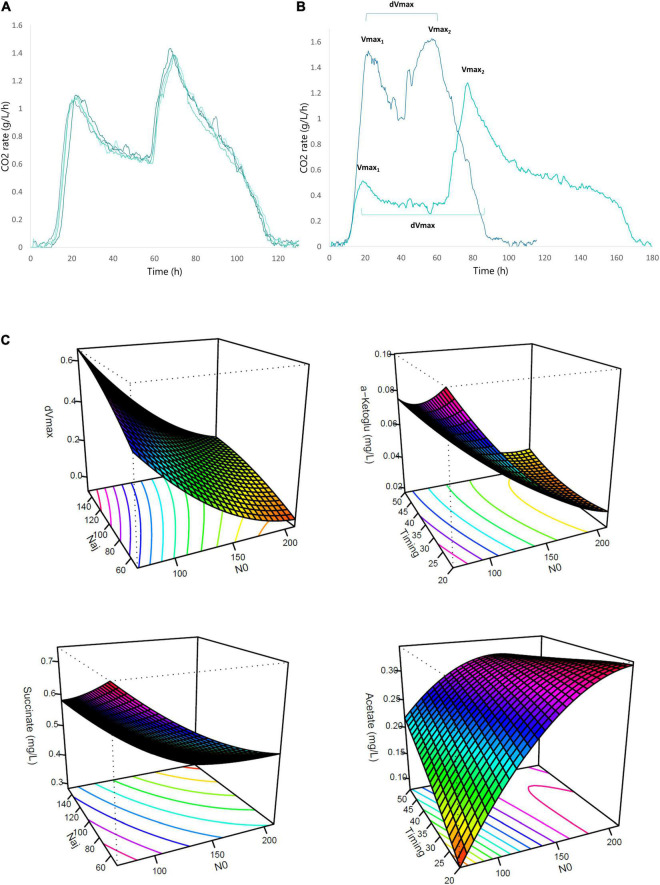
Effects of timing of nitrogen addition on fermentation kinetics and CCM metabolites. **(A)** Reproducibility of the fermentation kinetics of the Box-Behnken central point (SM140 with an addition of 100 mgN/L at 35 g/L of CO_2_ released). **(B)** Representation of the differences in fermentation kinetics between two fermentations with different initial and added nitrogen concentrations: MS210 with an addition of 50 mgN/L (dark blue curve) and MS70 with an addition of 150 mgN/L (light blue curve). **(C)** Response surface representing the impact of nitrogen addition timing on kinetic parameters (dVmax) and CCM metabolites (acetate, succinic acid, a-ketoglutarate) [NO (mg/L); Nad (mg/L); Tm (g/L CO_2_ released)].

A response surface model was used on the three factors with a Box-Behnken design (Table 1). The repetition of the four experiments corresponding to the central point of the design makes it possible to estimate the repeatability of the experiments. The fermentation kinetics obtained for these experiments were reproducible (Figure 1A) as were also cell counts (8.88.10^7^ ± 0.80.10^7^ cells/mL). The analysis showed a good reproducibility and non-significant lack-of-fit tests at a 5% threshold as well as great adjusted R^2^ thus allowing us to interpret the various models fitted.

**TABLE 1:** Experimental conditions for the 16 fermentations.

Experiments	Initial nitrogen (N0), mg/L	Timing of addition (Tm), CO_2_ g/L	Nitrogen added (Nad), mg/L
1	210	35	150
2	210	50	100
3	210	20	100
4	140	20	150
5	140	50	150
6^1^	140	35	100
7^2^	140	35	100
8^3^	140	35	100
9^4^	140	35	100
10	210	35	50
11	140	20	50
12	140	50	50
13	70	35	150
14	70	20	100
15	70	50	100
16	70	35	50

*1–4 Four replicates included at the center of the experimental domain.*

To evaluate the impact of the three studied parameters, we calculated different kinetic parameters: the first maximum CO_2_ production rate (Vmax1), the recovery rate after addition (Vmax2) and the total fermentation time (Figure 1B). Nevertheless, to evaluate the efficiency of nitrogen additions during the stationary phase, we calculated additional parameters. The first parameter was “dVmax,” i.e., the difference between the values of Vmax1 and Vmax2 (Figure 1B). The second parameter was “dPop,” the difference in population before and after obtaining the second maximum rate of CO_2_ production.

At first, we observed that Vmax1 increased with the initial nitrogen concentration (Table 2), as already shown in previous studies (Bely et al., 1990a; Mendes-Ferreira et al., 2004). Vmax1 was 0.5 g/L.h for an initial nitrogen concentration of 70 mg/L, while it reached 1.5 g/L.h for a 210 mg/L concentration (Figure 1B). Obviously, Tm and Nad had no effect on Vmax1 as nitrogen additions were systematically made during the stationary phase, i.e., after the first maximum CO_2_ production rate.

**TABLE 2:** Effects of three factors described by the model on fermentation kinetics and CCM metabolites.

	Simple effects			Interactions			Quadratics effects		
	N0	Tm	Nad	N0:Tm	N0:Nad	Tm:Nad	N0^2^	Tm^2^	Nad^2^
Fermentation time	−	−	+	+		−		−	
Vmax1	+								
dVmax	−	−	+	−	−	−	+		−
dPop	−	−					+		
Acetate	+			−			−		
α-ketoglutarate	−						+		
Succinate	−		−						

*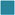 p.v < 0.001, 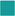 p.v < 0.01, 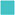 p.v < 0.05.*

On the opposite, the two other kinetic parameters calculated were statistically impacted by the three factors studied in this work. For the fermentation time and dVmax (Table 2), the model predicted a negative effect of N0 and Tm but a positive effect of Nad. For example, a high nitrogen concentration at the beginning of the fermentation resulted in a very high Vmax1, so that Vmax2 was low leading to a low dVmax (Table 2 and Figure 1B). Moreover, a late addition of nitrogen resulted in a low Vmax2 and so in a low dVmax (Table 2). Finally, Nad also had a very significant and positive influence on dVmax. Indeed, when the quantity of added nitrogen was high, the difference in velocity was high (Table 2 and Figure 1C). These comments also applied to fermentation time.

The concentration of initial nitrogen, added nitrogen and the timing of addition had important effects on fermentation kinetics and thus on the activation of yeast metabolism. This activation included effects on yeast growth and therefore on dPop. The effects of N0 and Tm on dPop were identical to the ones observed for dVmax (Table 2). On the contrary, the effect of Nad on dPop was very different; dPop was not impacted by this parameter in the range tested in this work. Finally, an addition carried out at the beginning of the stationary phase (24% of fermentation progress) resulted in cell growth, whereas no growth resumption was observed for additions carried out close to the end of the fermentation.

Glycerol production was similar under all conditions. However, there were production differences for α-ketoglutarate, succinic acid and acetic acid. For α-ketoglutarate, the model described a very significant and negative linear effect of N0 (Table 2 and Figure 1C). For succinic acid, negative effects were noted for both N0 and Nad. Finally, for acetic acid, the model described a very significant and positive effect of N0 (Table 2 and Figure 1C).

### Impact of Nitrogen Addition on Volatile Compound Production

The data presented for aroma compounds were those for which we had the most interpretable results. In addition, we had chosen these compounds because they are representative of different aroma families. So, we analyzed the production of four alcohols, two acetate esters, one acid and its corresponding ethyl ester (Table 3). Among these compounds, the three esters are involved in fruity flavor of young wines; so their study is interesting for a possible management of aroma production wines. Despite the lack of interest from an aromatic point of view, the study of the four alcohols is important for a better understanding of yeast metabolism.

**TABLE 3:** Effects of three factors described by the model on aroma production.

	Simple effects			Interactions			Quadratics effects		
	N0	Tm	Nad	N0:Tm	N0:Nad	Tm:Nad	N0^2^	Tm^2^	Nad^2^
Propanol		+	+			+		−	−
Isoamyl alcohol	−			+		+			
Isobutanol		−		+			−		
Phenyl ethanol	+					+	−		
Isoamyl acetate		−	+						
Isobutyl acetate	+	−	+						
Hexanoic acid	+	−		−		−			
Ethyl hexanoate				+					
Isoamyl ratio	+	−	+			−		−	
Hexanoic ratio	−	+	+	+		+		+	

*$I\,s\,o\,a\,m\,y\,l\,r\,a\,t\,i\,o=\frac{I\,s\,o\,a\,m\,y\,l\,a\,c\,e\,t\,a\,t\,e}{I\,s\,o\,a\,m\,y\,l\,a\,l\,c\,o\,h\,o\,l}\,H\,e\,x\,a\,n\,o\,i\,c\,r\,a\,t\,i\,o=\frac{E\,t\,h\,y\,l\,h\,e\,x\,a\,n\,o\,a\,t\,e}{H\,e\,x\,a\,n\,o\,i\,c\,a\,c\,i\,d}$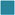 p.v < 0.001, 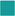 p.v < 0.01, 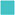 p.v < 0.05.*

#### Propanol

**FIGURE 2:**
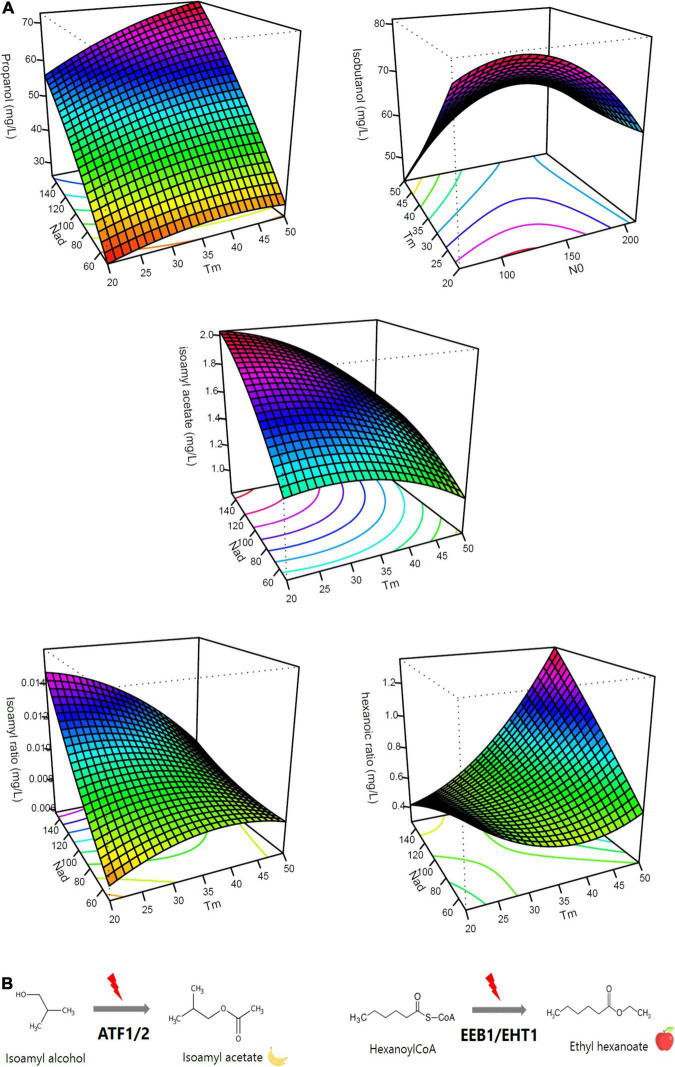
Effects of timing of nitrogen addition on aroma production. **(A)** Response surface representing the impact of nitrogen addition timing on aroma productions (propanol, isoamyl alcohol, isoamyl acetate, isoamyl ratio, and hexanoic ratio) [NO (mg/L); Nad (mg/L); Tm (g/L CO_2_ released)]. **(B)** Diagram representing the conversion of isoamyl alcohol to isoamyl acetate via the ATF1 and ATF2 enzymes and the conversion of hexanoyl-CoA to ethyl hexanoate via EEB1 and EHT1 enzymes.

At first, it should be noted that the impact of nitrogen additions on propanol production was very different from the one observed for the other alcohols, as already mentioned in the literature (Mouret et al., 2014b; Rollero et al., 2016; Seguinot et al., 2018). The production of propanol was only influenced by the factors Nad and Tm that both had a positive and very significant effect (Table 3). For example, for N0 = 70 mg/L and Nad = 100 mg/L, we observed a 26% increase for an addition at 50 g/L of CO_2_ released (56 mg/L) compared to an addition at 20 g/L (41 mg/L). In parallel, for N0 = 140 mg/L and Tm = 20 g/L, a 50% increase was noted for an addition of 150 mg/L (55 mg/L) compared to an addition of 50 mg/L (27 mg/L) (Table 4). This result indicated that a late nitrogen addition in high concentration favored the production of this compound (Figure 2A). Furthermore, we observed an interaction effect between Nad and Tm.

**TABLE 4:** Final concentration of volatile compounds (mg/L).

N0	Tm	Nad	Propanol (mg/L)	Phenyl ethanol (mg/L)	Isobutanol (mg/L)	Isobutyl acetate (mg/L)	Isobutyl ratio (mg/L)	Isoamyl alcohol (mg/L)	Isoamyl acetate (mg/L)	Isoamyl ratio (mg/L)	Hexanoic acid (mg/L)	Ethyl hexanoate (mg/L)	Hexanoic ratio (mg/L)
210	35	150	67.6	99.6	53.4	0.152	0.00284	103.4	1.66	0.0160	1.651	0.818	0.496
210	50	100	53.5	106.7	68.2	0.160	0.00234	162.9	1.88	0.0116	1.023	0.908	0.887
210	20	100	45.8	82.2	62.1	0.164	0.00264	129.1	1.82	0.0141	2.472	0.726	0.294
140	20	150	55.5	85.0	70.7	0.192	0.00271	141.6	2.00	0.0141	1.708	0.743	0.435
140	50	150	70.8	125.0	59.3	0.101	0.00170	150.0	1.29	0.0086	0.502	0.687	1.367
140	35	100	51.9	109.6	73.1	0.181	0.00248	179.9	1.87	0.0104	0.932	0.695	0.746
140	35	100	49.7	119.5	73.4	0.142	0.00193	169.6	1.73	0.0102	1.054	0.709	0.673
140	35	100	52.8	117.4	68.0	0.145	0.00213	161.4	1.62	0.0100	1.225	0.741	0.605
140	35	100	49.6	98.4	64.6	0.142	0.00220	152.3	1.51	0.0099	1.345	0.687	0.511
210	35	50	32.2	101.0	59.3	0.134	0.00227	132.3	1.44	0.0109	1.915	0.675	0.353
140	20	50	27.7	122.7	84.5	0.126	0.00150	206.1	1.45	0.0070	1.029	0.778	0.756
140	50	50	29.7	97.7	52.7	0.095	0.00179	132.4	1.10	0.0083	1.177	0.692	0.588
70	35	150	68.6	79.6	60.8	0.147	0.00241	171.5	2.04	0.0119	1.035	0.769	0.743
70	20	100	41.2	83.5	72.6	0.103	0.00142	195.8	1.49	0.0076	1.034	0.834	0.807
70	50	100	56.5	80.4	45.3	0.068	0.00150	140.7	0.75	0.0053	0.597	0.568	0.952
70	35	50	26.9	73.9	53.3	0.074	0.00139	155.5	0.90	0.0058	1.133	0.677	0.598
**Central point of the model**													
Average			51.0	111.2	69.8	0.153	0.00219	165.8	1.68	0.0101	1.139	0.708	0.634
Standard error			1.6	9.6	4.2	0.019	0.00023	11.7	0.153	0.0002	0.182	0.024	0.100
Coefficient of variation (%)			3.14	8.6	6.08	12.6	10.4	7.08	9.08	2.01	16	3.37	15.8

#### Higher Alcohols

For the three higher alcohols analyzed (isoamyl alcohol, isobutanol, and phenylethanol), the influence of nitrogen additions was different (Table 3). No effect of Nad was noted for isoamyl alcohol and isobutanol. For isoamyl alcohol, a negative linear effect of N0 was observed. There was also a positive interaction effect between N0:Tm and Tm:Nad. Indeed, for N0 = 210 mg/L and Nad = 100 mg/L, a 20% increase was observed for an addition at 50 g/L of CO_2_ released (162 mg/L) compared to an addition at 20 g/L (129 mg/L) (Table 4). With regard to isobutanol production, a negative linear effect of timing was observed. For N0 = 70 mg/L and Nad = 100 mg/L, we noted a 37% decrease for a late addition (45 mg/L) compared to an early addition (72 mg/L) (Table 4). Moreover, we observed an interaction effect between N0 and Tm and a negative quadratic effect of N0 (Table 3 and Figure 2A).

Finally, for phenylethanol, at a threshold of 5%, we noted a significant and positive interaction between the timing of addition and nitrogen added as well as a positive effect of the initial nitrogen. This data indicated that the maximum content of this compound was reached for a high and late addition of nitrogen. We observed a 32% increase for a late addition compared to an early addition for the same concentrations of initial (N0 = 140 mg/L) and added nitrogen (Nad = 150 mg/L) (Table 4).

#### Acetate Esters

The production of the two acetate esters studied was impacted by the three fermentation parameters in nearly the same way. We note that the model described only linear effects. Isoamyl acetate was negatively influenced by Tm but positively by Nad; isobutyl acetate showed the same effects with an additional positive effect of N0 (Table 3). This data indicated that the production of acetate esters was favored with an early addition of nitrogen in high concentration (Figure 2A). For N0 = 140 mg/L, we observed an increase of 27 and 34%, respectively, for an early addition of 150 mg/L compared to an early addition of 50 mg/L (Table 4).

#### Fatty Acid and Ethyl Ester

For hexanoic acid, the model described both linear and interaction effects. The production of this acid was positively impacted by N0 and negatively by Tm. For N0 = 210 mg/L and Nad = 100 mg/L, we observed a 58% increase for an early addition (2.47 mg/L) compared to a late addition (1.02 mg/L) (Table 4). There was also an interaction effect between these two factors and between Tm and Nad. By contrast, the production of the corresponding ester, ethyl hexanoate, did not present the same profile at all, as there was only a positive interaction effect between N0 and Tm (Table 3), indicating that the effect of the timing depended on the initial nitrogen concentration.

#### Ratio Between Esters and Their Precursors

To better understand the effect of nitrogen additions on the production of acetate esters and ethyl esters, we studied the ratios between the production of these esters (acetate ester and ethyl ester) divided by the production of their precursors (i.e., the corresponding higher alcohol or acid, respectively).

For the ratio isoamyl acetate/isoamyl alcohol (Table 3), we noted that N0 and Nad factors had significant and positive linear effects while the Tm factor had a negative one (Table 3). These results indicated that a greater conversion of higher alcohols into esters (Figure 2B) was observed for a high initial nitrogen concentration and a high amount of added nitrogen (Figure 2A). For N0 = 210 mg/L and Tm = 35 g/L, we observed a 32% increase for a nitrogen addition of 150 mg/L compared to an addition of 50 mg/L (Table 4). Furthermore, an early addition seems to maximize this ester/alcohol ratio (increase of 30% for an early addition compared to a late addition of 100 mg/L of nitrogen and N0 = 70 mg/L) (Figure 2A).

Looking at the ratio between ethyl hexanoate and hexanoic acid, it can be seen that all three factors had significant linear effects but that these effects were very different from those of the ratio between an acetate ester and a higher alcohol. For the ratio between ethyl hexanoate and hexanoic acid, N0 had a negative effect. Conversely, the Tm and Nad factors had a significant and positive linear effect (Table 3). In addition, there were very significant interaction effects between the Nad and Tm factors as well as a quadratic effect of the Tm factor (Figure 2A). We observed a 66% increase for a late addition (0.88) compared to an early addition (0.29) for N0 = 210 mg/L and Nad = 100 mg/L (Table 4). This result indicated that the conversion (Figure 2B) increased for a late nitrogen addition at a high level (Figure 2A).

### Effects of Nitrogen Additions on Gene Expression

To better understand the results obtained thanks to the Box-Behnken model, we compared the overall expression patterns by microarray analyses using three biological replicates. The impact of the timing of nitrogen addition on the expression of all yeast genes was studied. It can be observed that 17% of total genes were differentially expressed.

**FIGURE 3:**
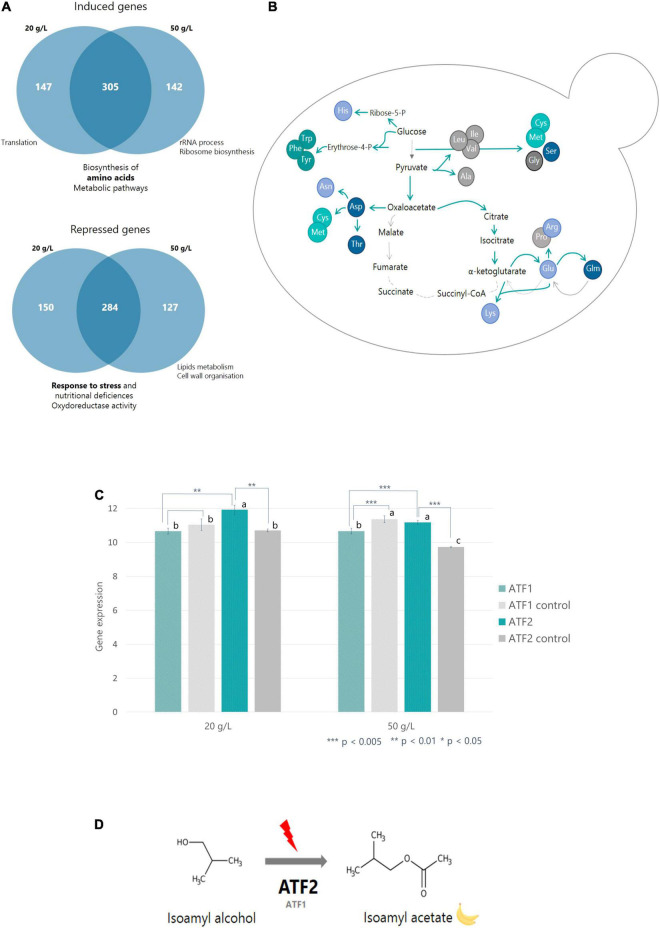
Effects of timing of nitrogen addition on gene expression. **(A)** Venn diagram representing the groups of genes induced or repressed after nitrogen addition and their associated functions. Functions were determined using GeneCodis. **(B)** Scheme representing the activation/repression of CCM and biosynthesis of amino acid genes following nitrogen addition. Blue arrows represent steps genetically induced by nitrogen additions; gray arrows are steps repressed by nitrogen additions. **(C)** Histograms representing the expression of *ATF1* and *ATF2* genes following nitrogen addition performed at 20 g/L or 50 g/L CO_2_ released. *ATF1/2* controls were performed on fermentations without nitrogen addition. a, b and c are ANOVA groups that correspond to significantly different gene expression values. **(D)** Diagram representing the conversion of isoamyl alcohol to isoamyl acetate via ATF2 and ATF1 following nitrogen addition.

Venn diagrams were created to see which cellular functions were induced or repressed by nitrogen addition and to evaluate the impact of the addition timing. The first diagram represents induced genes and the second repressed genes (Figure 3A). In the first diagram, 51% of induced genes were common to both addition timings and represented amino acid biosynthetic functions and metabolic pathways. 25% of genes were induced after an addition at the beginning of the stationary phase and 24% exclusively after an addition at the end of the stationary phase. These genes corresponded to translation and ribosomal RNA synthesis functions, respectively.

In the second diagram, 28% of genes involved in stress response and nutritional deficiencies as well as oxidoreductase activity were repressed after an addition at the beginning of the stationary phase. The genes common to both timings (50%) are involved in the same functions. Finally, 22% of genes repressed after an addition close to the end of the fermentation were genes involved in lipid metabolism and cell wall organization (Figure 3A).

#### Impact of Nitrogen Addition Timing on Gene Expression Coding for Proteins Involved in the Pathway of Central Carbon Metabolism and Biosynthesis of Amino Acids

To understand in more detail the effect of nitrogen addition on yeast metabolic fluxes, the expression of genes coding for the enzymes involved in glycolysis, the Krebs cycle and amino acid synthesis was examined.

After nitrogen addition and whatever its timing, the expression of the genes encoding glycolysis enzymes did not change, suggesting that the glycolytic flux was permanently activated. On the other hand, regarding the Krebs cycle enzymes, the genes encoding pyruvate synthesis up to α-ketoglutarate (*PYC1*, *PYC2*, *CIT2*, and *ACO2*) were overexpressed (Figure 3B). Expression of *GDH1* and *GDH3* genes, encoding the enzymes responsible for glutamate synthesis, also increased significantly. Conversely, expression of genes involved in succinate, fumarate, and malate biosynthesis (*GDH2*, *KGD1-2*, *LSC1-2*, *SDH1-4*, and *MDH1-2*) was repressed (Figure 3B).

#### Impact of Nitrogen Additions on Expression of Genes Encoding Proteins Involved in the Pathway of Volatile Compounds

In this work, we observed that the addition of nitrogen enabled the activation of metabolic flux toward amino acid synthesis. The resulting amino acids can also be converted to aldehydes and then to higher alcohols via ketoacids (Bloem et al., 2016). The formation of aldehydes from ketoacids is catalyzed by the family of aminotransferases (ARO enzymes). The *ARO8* gene, coding for the enzyme responsible for the conversion of phenylalanine into phenylpyruvate, was induced by nitrogen addition regardless of the timing of addition. The formation of aldehydes can also be catalyzed by the enzymes encoded by the *PDC1* and *PDC5* genes. The expression of these two genes as well as that of the genes coding for the ADH family of enzymes (formation of higher alcohols from aldehydes) was not impacted by the addition of nitrogen. However, one gene of this ADH family, the ADH5 gene, was induced by the addition of nitrogen regardless of the time of addition (20 or 50 g/L of released CO_2_).

Then, we looked at the *ATF1* and *ATF2* genes, coding for the enzymes responsible for the formation of acetate esters from higher alcohols (Figure 3C). While *ATF1* expression was not modified by nitrogen addition, *ATF2* gene was overexpressed in the two conditions, with a higher increase in addition realized at the beginning of the stationary phase (Figure 3C).

Finally, the expression of the *EEB1* and *EHT1* genes coding for the enzymes responsible for the conversion of acids to ethyl esters was also examined but no effect of nitrogen addition was observed.

## Discussion

The aim of our study was to better understand the impact of nitrogen addition during stationary phase on yeast metabolism and especially on fermentative aromas synthesis. To reach this goal, we combined the use of statistical modeling and transcriptomic analysis.

### Cell Population and Central Carbon Metabolism

We first looked at the effects of nitrogen addition during the stationary phase on growth and the main fermentation kinetics. Following any nitrogen addition, this nutrient was quickly and entirely consumed to produce amino acids, as indicated by transcriptomic analyses showing that the genes coding for enzymes involved in amino acids biosynthesis were systematically overexpressed whatever the timing of nitrogen addition. Nevertheless, our results indicated that an early nitrogen addition induced an increase in the cell population whereas this phenomenon was not observed for a late addition, as already observed by Malherbe et al. (2004). This result suggested that added nitrogen was then either used for the resumption of cell growth or for the production of proteins, such as sugar transporters as shown in the literature (Salmon, 1989; Bely et al., 1994). These two effects led to an increase in fermentation rate that was observed under both addition conditions. However, the efficiency of these additions was different: an early nitrogen addition was more effective because it was associated with growth. This suggested that the resumption of growth allows for an increase in Vmax in addition to a mere stimulation of cell activity. In relation to this effect on growth, the timing of nitrogen addition also affected the effectiveness of the increase in fermentation rate. This rise in CO_2_ production rate was higher for an earlier nitrogen addition but also for higher concentrations of added and initial nitrogen (Table 2). This directly impacted the fermentation time that was significantly reduced following nitrogen addition during the stationary phase. This last result confirms the fact that the efficiency on fermentation time is equivalent for additions made during the first half of the stationary phase (Malherbe et al., 2004).

This consumption of nitrogen during the stationary phase also impacted the central carbon metabolism and in particular the production of metabolites originating from the Krebs cycle, as already observed by Barbosa et al. (2015). In the present work, gene expression analysis showed that all genes responsible for pyruvate synthesis up to α-ketoglutarate synthesis were overexpressed following nitrogen addition. By contrast, this analysis showed that the genes responsible for succinate, fumarate and malate synthesis were repressed. Thus, consequently to a nitrogen addition during the stationary phase, the flux of the oxidative branch of the Krebs cycle is higher than the one of the reductive way, as previously observed during the growth phase (Camarasa et al., 2003). These genetic regulations can explain the results obtained for the production of succinate and α-ketoglutarate (Table 2 and Figure 1C). On one hand, succinate production was negatively impacted by added nitrogen concentrations. On the other hand, the production of α-ketoglutarate did not depend on the timing of nitrogen addition or the concentration of added nitrogen. These observations show that, following nitrogen addition during the stationary phase, the pool of α-ketoglutarate is mainly redirected to amino acid synthesis at the expense of succinate synthesis. These findings are supported by the results of the transcriptomic analysis. Indeed, following nitrogen addition (regardless of the addition timing) the genes responsible for amino acid synthesis were all overexpressed. Finally, the surplus of amino acids produced is used either for the resumption of cell growth (Ljungdahl and Daignan-Fornier, 2012) or for protein turnover depending of the timing of nitrogen addition (as presented in the previous paragraph related to biomass synthesis).

For glycerol production, we found no significant effect of the three factors tested in this study. Furthermore, gene expression analysis showed that the genes responsible for glycerol synthesis are repressed following nitrogen addition, regardless of the timing of the addition. Several studies have shown that initial nitrogen concentration either does not impact glycerol concentration or induces a decrease in this compound (Beltran et al., 2005; Martínez-Moreno et al., 2014; Rollero et al., 2015; Gobert et al., 2019; Guittin et al., 2021). However, in these previous works, high nitrogen contents were used and nitrogen addition consisted in a mixture of ammonium and amino acids. In the present work, we only added ammonia nitrogen during the stationary phase and in lower concentrations. These two factors appear to be the reason for the differences between our results and data found in the literature.

Finally, acetate production was only impacted by initial nitrogen content and not by nitrogen added during the stationary phase. Acetate is involved in (NADPH) redox balance especially during growth. This compound is mainly synthesized from acetaldehyde by the enzyme Ald6p but the mitochondrial isoform Ald5p can also contribute to its production (Saint-Prix et al., 2004). *ALD5* gene is overexpressed following nitrogen addition during the stationary phase. So, it could be hypothesized that, after this nutrient supplementation, the acetate pool is mainly mobilized toward acetyl-CoA synthesis (Swiegers et al., 2005) for its incorporation into the oxidative branch of the Krebs cycle (Saint-Prix et al., 2004) to meet yeast requirements in terms of precursors or redox balance. Its consumption for yeast metabolism was very high and did not result in any extracellular excretion.

### Volatile Compound Synthesis

The effect of nitrogen addition differs depending on the volatile compounds.

Propanol production appeared dependent on added nitrogen and timing in a positive way but was independent of the initial nitrogen level (Table 3). Such an observation was surprising because a linear correlation between initial nitrogen content and final propanol concentration had previously been noted in different research works (Carrau et al., 2008; Moreira et al., 2011; Mouret et al., 2014b; Rollero et al., 2015; Seguinot et al., 2018). This observation was probably due to the fact that nitrogen addition during the stationary phase had a much higher efficiency than a high initial nitrogen content. This last hypothesis was in agreement with the results obtained by Seguinot et al. (2018), which indicate that, for a given quantity of added ammonium, the final propanol concentration is 2.5 times higher when the nitrogen supplementation is done during the stationary phase compared to an addition at the start of the fermentation process. So, the data presented in this research work confirm that propanol is a marker of amount and timing of assimilable nitrogen addition in the must (Mouret et al., 2014a; Seguinot et al., 2018).

We have shown that a late addition of nitrogen induced an increase in propanol production. We have also shown that a late addition of nitrogen allows the reactivation of cell activity but is not associated to yeast regrowth. α-ketobutyrate is the common precursor of propanol and isoleucine. It has been suggested that an addition of nitrogen during the stationary phase would redirect the α-ketobutyrate pool toward propanol synthesis but not isoleucine synthesis (Seguinot et al., 2018). This hypothesis is consistent with our results since, in our case, propanol synthesis is maximized after late nitrogen addition. Indeed, it can be hypothesized that, since amino acids are no longer used for growth, part of the flux could be redirected toward α-ketobutyrate synthesis via transamination and then to propanol synthesis.

The production of phenylethanol is impacted differently from isoamyl alcohol and isobutanol. Its production is favored by a late addition of nitrogen and by a high concentration of initial nitrogen. The synthesis of this compound depends on the pentose phosphate pathway and in particular on phenylalanine via the enzyme ARO8 (Ljungdahl and Daignan-Fornier, 2012). This enzyme is an aminotransferase and allows either the synthesis of phenylalanine or the synthesis of phenylpyruvate (Natarajan et al., 2001). In our study, the *ARO8* gene is overexpressed after nitrogen addition. In the same way as propanol, for a late addition of nitrogen, since amino acids are not used by the yeast regrowth, it is possible that part of the phenylalanine synthesis flux is directed to the synthesis of phenylpyruvate via ARO8 and thus of phenylethanol.

As already observed in previous studies (Mouret et al., 2014b; Rollero et al., 2015), isobutanol and isoamyl alcohol behaviors were very different compared to propanol. The synthesis of these two higher alcohols was only weakly impacted by nitrogen addition during the stationary phase, as previously noted by Beltran et al. (2005) and Seguinot et al. (2018). This very limited effect could be explained by the fact that α-ketoacids that are isobutanol and isoamyl alcohol precursors are mainly produced from the central carbon metabolism and that the contribution to their synthesis of the amino acids catabolism (by the Ehrlich pathway) is minor (Crépin et al., 2017; Rollero et al., 2017). Therefore, it could be suggested that following nitrogen addition, the supplementary flux of alpha-ketoacids related to the Ehrlich pathway remained negligible compared to the flux from CCM and that the overall isobutanol and isoamyl alcohol production did not change. Moreover, *ADH5* gene expression was increased following nitrogen addition. *ADH5* is responsible for the synthesis of higher alcohols from α-ketoacids (Dickinson et al., 2003). However, it has been shown that the enzyme Adh5p can perform the reverse reaction, i.e., α-ketoacids synthesis from higher alcohol (de Smidt et al., 2008). This could explain that isobutanol production was not impacted by nitrogen addition while isoamyl alcohol production was negatively impacted. The flux would go to α-ketoacids and then to amino acids and would support the previous results.

Concerning acetate esters, our data confirmed the positive influence of nitrogen addition on their production (Mouret et al., 2014a; Seguinot et al., 2018; Su et al., 2021; Figure 2B). Nevertheless, results obtained in the present work also provided additional knowledge: acetate ester production was mainly controlled by the timing and level of nitrogen addition during the stationary phase. For isoamyl acetate, the weight of these two factors was so considerable that it masked the positive effect of a high initial nitrogen content usually observed in previous studies (Mouret et al., 2014a; Rollero et al., 2015). Finally, the fact that higher alcohols and acetate esters were very differently impacted by nitrogen additions indicated that the key element in the production of these esters was enzymatic activity and not precursors availability.

Unlike acetate esters, no direct effect of the three parameters studied in the present work was observed for ethyl esters. This observation confirmed that nitrogen was not the most efficient nutrient to drive ethyl ester production, which is not very surprising as ethyl esters are related to lipid- and not nitrogen-metabolism (Bloem et al., 2016; Liu et al., 2018). Moreover, it should be noted that acid production was impacted by the initial nitrogen content and the timing of addition. Observing differences in the impact of the parameters on the synthesis of ethyl esters and their corresponding precursors suggested that acid availability is not the key element in the synthesis of ethyl esters, contrary to what was shown in previous studies (Saerens et al., 2008; Rollero et al., 2015). These differences can be attributed to the fact that, in these earlier works, nitrogen addition was made at the beginning of the fermentation and not during the stationary phase. Therefore, our results seem to indicate that regulation of this enzymatic activity is different during the growth and stationary phases.

In conclusion, our observations indicate that precursors availability is not the main factor in the synthesis of both acetate and ethyl esters. So, it can be thought that the enzymatic activity of precursors conversion into final products is the key element, especially consequently to an addition of nitrogen during the stationary phase. Therefore, the ratios between esters and their corresponding precursors was carefully studied.

The ratios between acetate esters and higher alcohols were positively impacted by initial and added nitrogen content but negatively by timing (Table 3 and Figure 3A). This result indicates that the synthesis of acetate esters mainly depends on the enzymatic activity responsible for the bioconversion of higher alcohols and not on their availability (Saerens et al., 2010; Seguinot et al., 2018). This hypothesis is supported by the transcriptomic analysis. It was shown that the *ATF2* gene was overexpressed following any nitrogen addition during the stationary phase; nevertheless, its expression level was higher for an addition at 20 g/L than for an addition at 50 g/L of released CO_2_. This supports the fact that an addition at the beginning of the stationary phase is more effective in increasing acetate ester synthesis. On the contrary, the expression of the *ATF1* gene (the other gene coding for the enzymatic conversion of higher alcohols into acetate esters) was not affected by nitrogen additions (Figure 3D). Such a result is surprising because it was previously established, through mutant construction, that the gene *ATF1* plays a major role in the synthesis of acetate esters while gene *ATF2* plays only a minor one (Verstrepen et al., 2003). Therefore, our data provide new information concerning the regulation of these two genes. This suggests that the expression of the *ATF1* gene is constitutive and not sensitive to nitrogen additions while that of the *ATF2* gene is inducible and responsive to environmental stimuli (Figure 3D).

The effects of the factors on ratios between ethyl esters and acids are very different from the acetate ratios. This indicates that the regulation of the two enzyme activities responsible for the synthesis of the two types of esters are very different: this is the first time that this information is shown. Conversely to acetate ratios, the ethyl ester/acids ratio was positively impacted by the timing and level of nitrogen addition. Thus, the conversion of acids to ethyl esters would be greater when nitrogen is added in high concentration in the second part of the stationary phase. In addition, it should be noted that the transcriptomic analysis showed that the expression of genes responsible for bioconversion of fatty acids into ethyl esters, i.e., *EEB1* and *EHT1*, was not impacted by the addition of nitrogen, regardless of the timing. All these results seem to indicate that the production of ethyl esters is regulated allosterically and not through gene expression.

## Conclusion

This study led us to better understand the effect of added nitrogen during the stationary phase in its entirety. To access a maximum of information on these phenomena, we developed a mathematical modeling approach to describe the effects of the studied factors and a complementary transcriptomic approach to explain some of our mathematical results. In the present work, it was demonstrated that an addition of nitrogen activated the central carbon metabolism and the biosynthesis of amino acids. It was also shown that the conversion of higher alcohols into acetate esters was maximized when nitrogen was added at the beginning of the stationary phase. New insight into the regulation of genes *ATF1*/*2* was given: *ATF1* gene expression would be constitutive and not sensitive to nitrogen additions while that of the *ATF2* gene would be inducible. Finally, it appears that the key element in the synthesis of both acetate and ethyl esters is the enzymatic activity responsible for their production, rather than the availability of their corresponding precursors. Nevertheless, consequently to nitrogen addition during the stationary phase, the regulation of ester production is different: for acetate esters, it is related to the expression level of the gene *ATF2* whereas for ethyl esters, it seems to be allosterically regulated.

For the wine industry, this work confirms the value of nitrogen addition during the fermentation process to better manage wine aroma production. Further work will be necessary but our data already demonstrate that the timing of addition and the quantity of added nitrogen strongly impact the final aroma composition of the wine.

Moreover, in the future, the data generated in this work will be helpful for the development of a more complex predictive model based on dynamic data in order to better manage the production of volatile compounds in wines.

## Data Availability Statement

The datasets presented in this study can be found in online repositories. The names of the repository/repositories and accession number(s) can be found below: https://www.ncbi.nlm.nih.gov/geo/, GSE189010.

## Author Contributions

MP and CP designed the experimental strategy to perform all fermentations of this study and developed the associated tools including automatic nitrogen addition. VG developed the transcriptomic analysis strategy. IS designed the Box-Behnken experimental design, participated in the development of the transcriptomic analysis with VG, and performed the statistical analysis of the Box-Behnken part and transcriptomic analysis. JG implemented all the experiment and drafted the manuscript. J-RM, VF, and JG performed the interpretation of all the data conceived and designed the overall study. J-RM and J-MS revised the manuscript. All authors read and approved the final version of the manuscript.

## Conflict of Interest

The authors declare that the research was conducted in the absence of any commercial or financial relationships that could be construed as a potential conflict of interest.

## Publisher’s Note

All claims expressed in this article are solely those of the authors and do not necessarily represent those of their affiliated organizations, or those of the publisher, the editors and the reviewers. Any product that may be evaluated in this article, or claim that may be made by its manufacturer, is not guaranteed or endorsed by the publisher.

## Footnotes

1
http://alfis.supagro.inra.fr/fermentation/

2
https://genecodis.genyo.es/
